# Supplementary material for: Stress-induced stenotic vascular remodeling via reduction of plasma omega-3 fatty acid metabolite 4-oxoDHA by noradrenaline
Source: Sci Rep. 2024 Feb 20;14:4178. doi: 10.1038/s41598-024-54867-3 (PMC10879168; doi:10.1038/s41598-024-54867-3)

Supplemental Figure 1

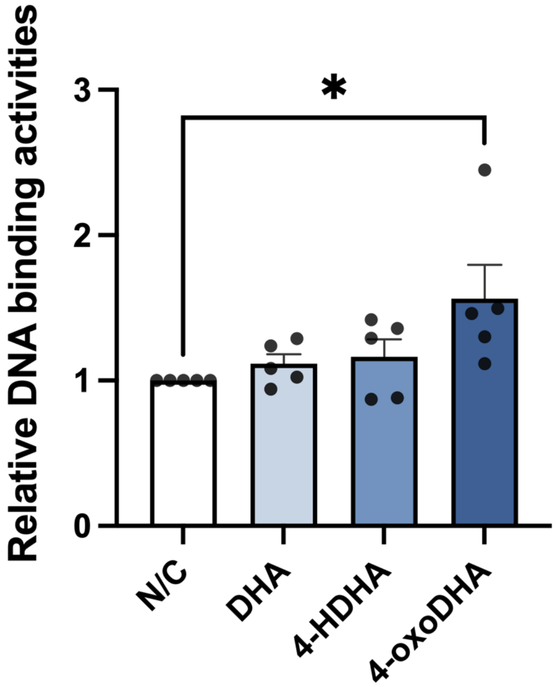

Supplementary Figure.S1.
RAW 264.7 cells were incubated with Kdo2 (0.5 μg/mL) for 1h, followed by treatment with 10 μM DHA, 4-HDHA, and 4-oxoDHA for 2h at 37℃. The DNA binding activity of Nrf2 was significantly augmented by 4-oxoDHA treatment. Data are expressed as fold induction of DNA-binding activity compared to vehicle (N/C), mean ± s.e. (n=5).
* indicates $p<0.05$.

Supplemental Figure 2

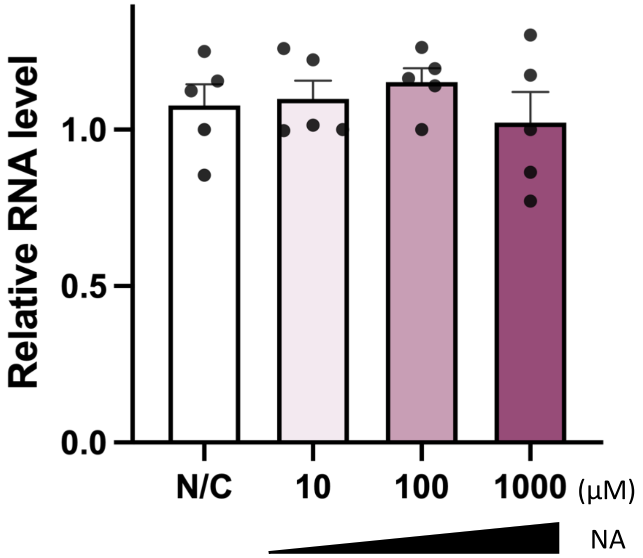

Supplementary Figure.S2.
HL-60 cells were treated with noradrenaline (10–1000 μM) for 2.5h at 37℃, and 5-LO mRNA expression levels were analyzed using real-time PCR. The 5-LO mRNA expression did not show any significant changes. Data are expressed as fold-change compared to vehicle (N/C), mean ± s.e. (n=5).

Supplemental Figure 3

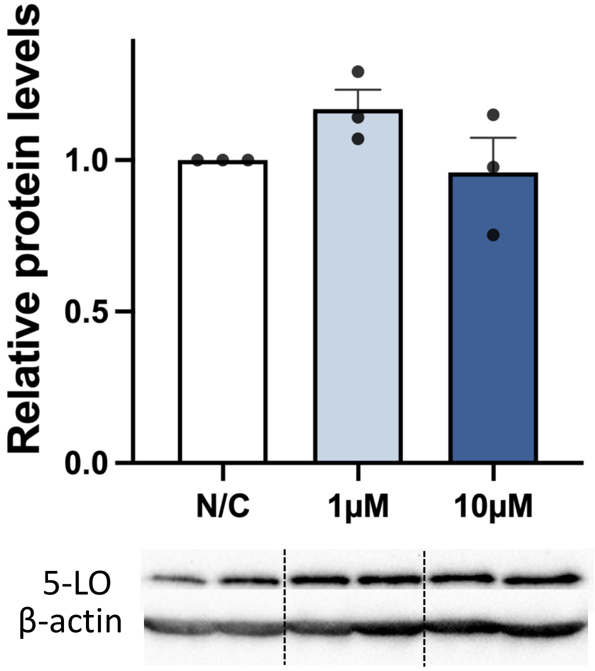

Supplementary Figure.S3.
HL-60 cells were treated with cortisol (1–10 μM) for 2.5h at 37℃, and 5-LO protein expression levels were analyzed using western blotting. The 5-LO protein expression did not show any significant changes. Data are expressed as fold-change compared to vehicle (N/C), mean ± s.e. (n=3).

Supplemental Figure 4

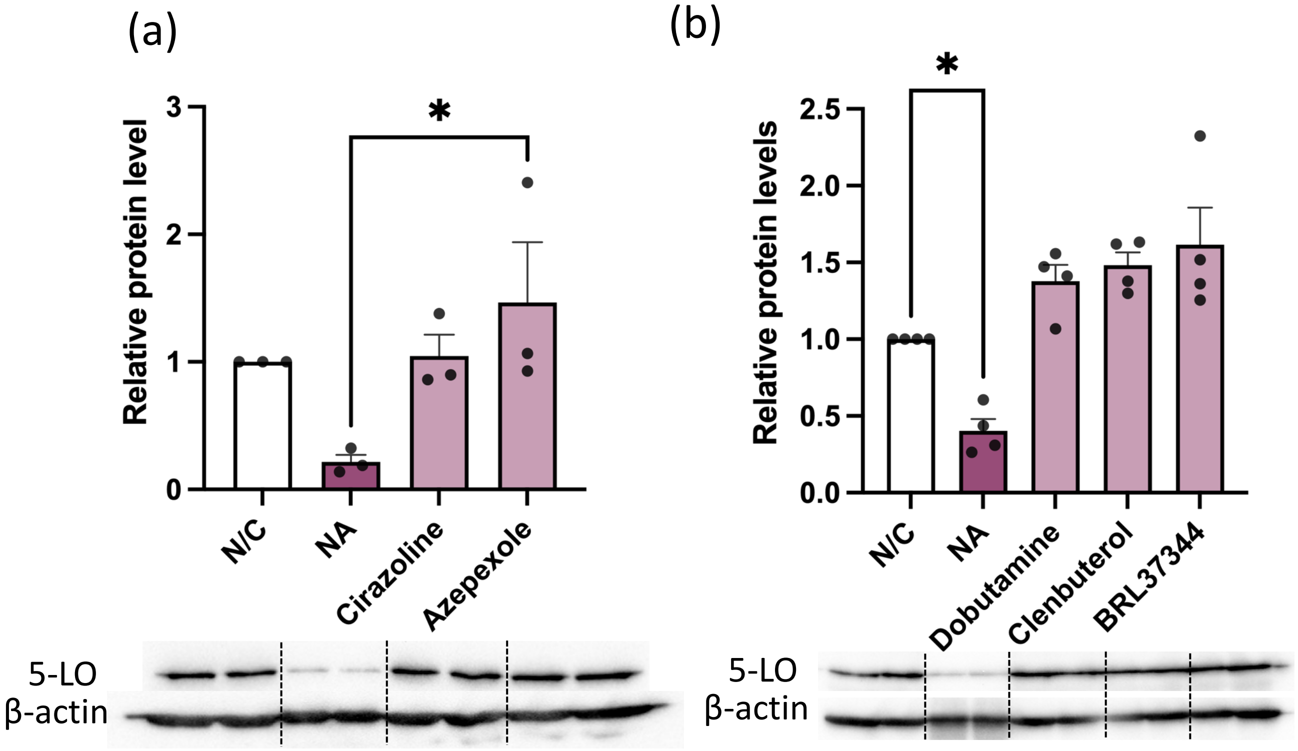

Supplementary Figure.S4.
HL-60 cells were treated with noradrenaline (1000 μM) and (a) selective adrenergic α receptor agonists (cirazoline and azepexole, 100 μM each) or (b) selective adrenergic β receptor agonists (dobutamine, clenbuterol, and BRL37344, 100 μM each) for 2.5h at 37℃, and 5-LO protein expression levels were analyzed using western blotting. Neither selective adrenergic α-receptor agonists (a) nor selective adrenergic β-receptor agonists (b) reduced 5-LO protein expression, whereas noradrenaline treatment significantly reduced 5-LO protein expression. Data are expressed as fold-change compared to vehicle (N/C) and mean ± s.e. (n=4).
* indicates $p<0.05$.

Supplemental Figure 5

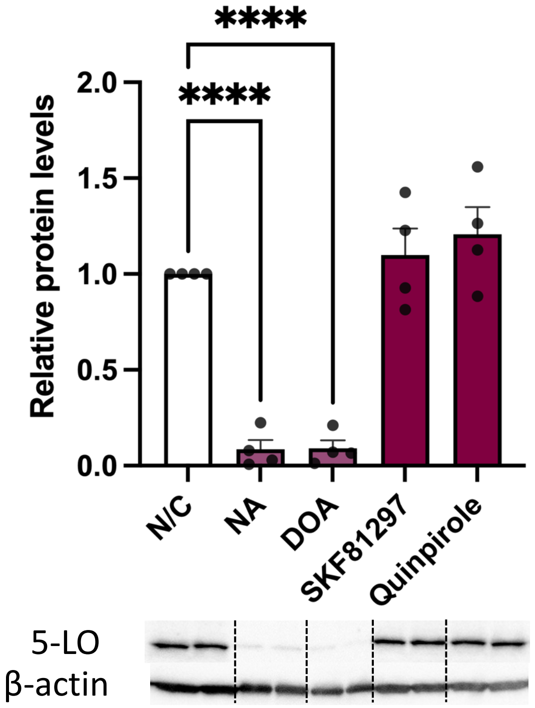

Supplementary Figure.S5.
HL-60 cells were treated with SKF81297 (D1-like receptor agonist, 10 μM) or quinpirole (D2-like receptor agonist, 10 μM) for 2.5h at 37℃, and 5-LO protein expression levels were analyzed using western blotting. Neither the D1-like receptor agonist nor the D2-like receptor agonist reduced 5-LO protein expression, whereas noradrenaline (1000 μM) or dopamine (DOA, 500 μM) treatment significantly reduced 5-LO protein expression. Data are expressed as fold-change compared to vehicle (N/C) and mean ± s.e. (n=4). **** indicates $p<0.0001$.

Supplemental Figure 6

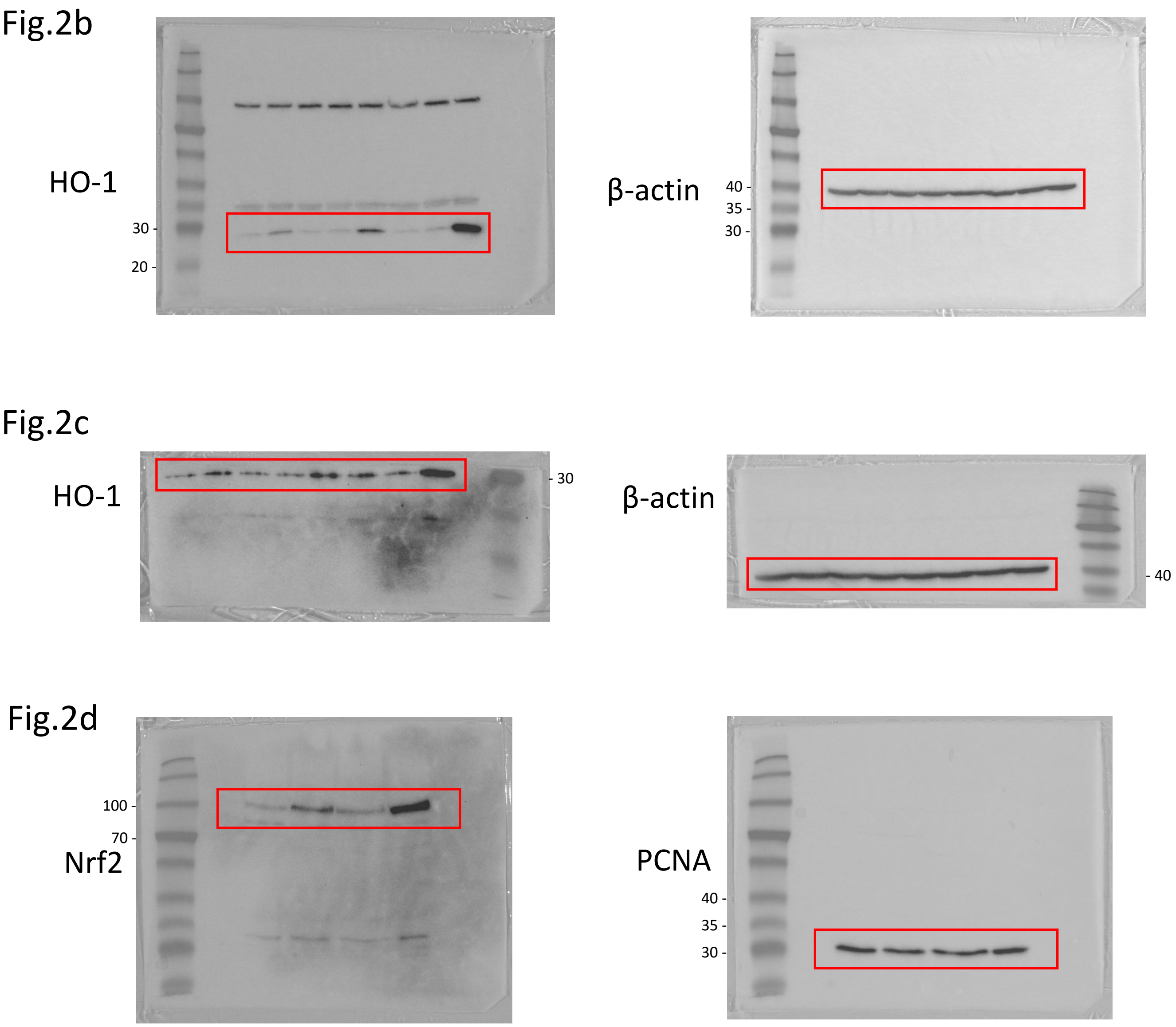

Supplemental Figure 7

Fig.3a

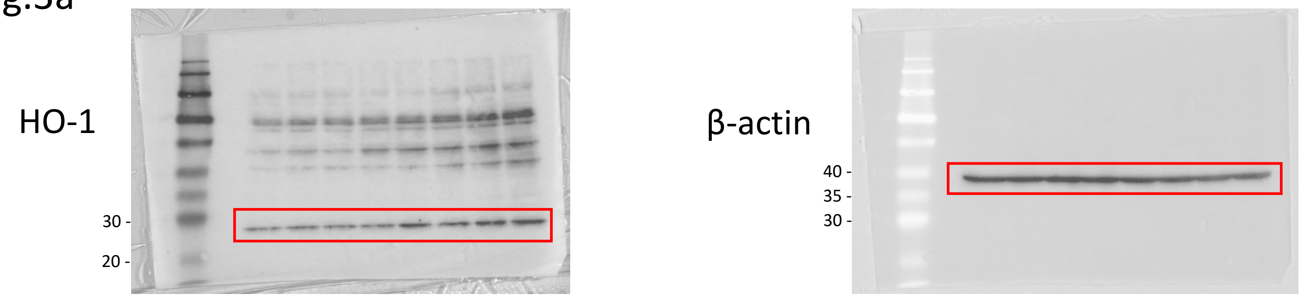

Fig.3b

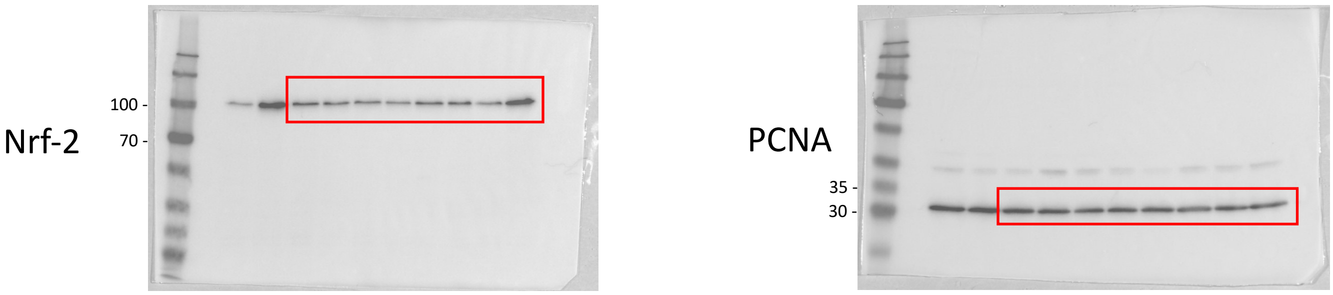

Fig.4c

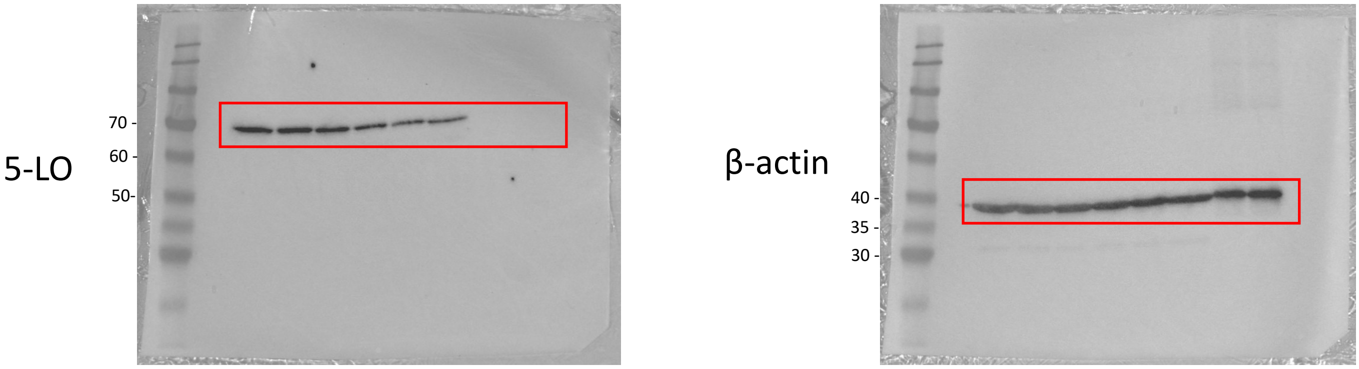

Supplemental Figure 8

Fig.4e

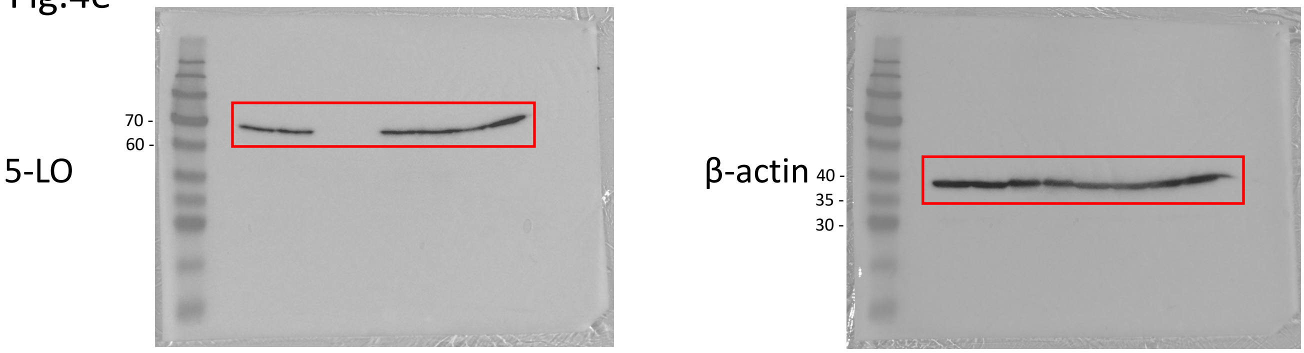

Fig.4f

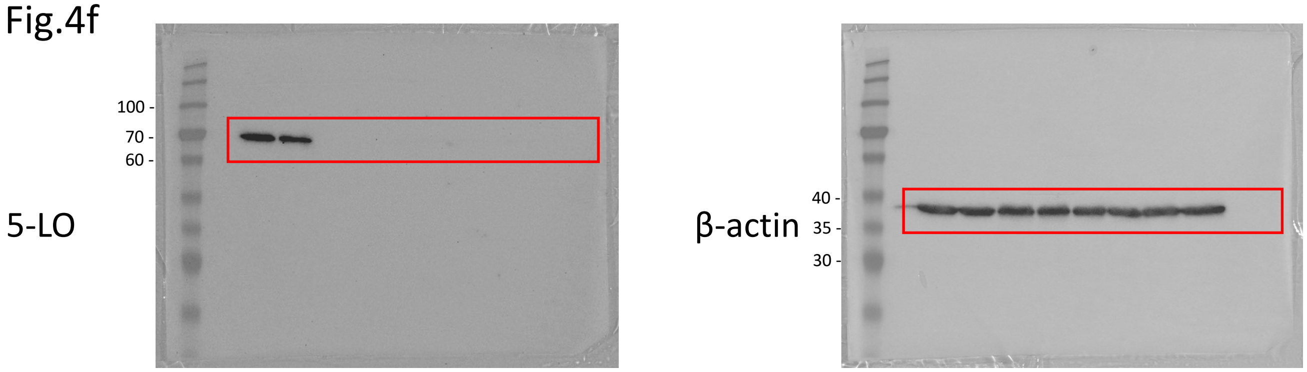

Fig.4g

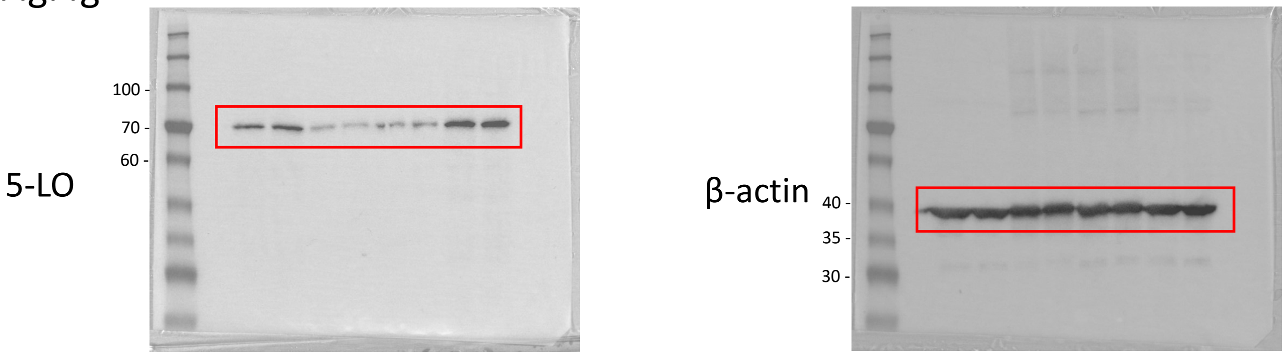

Supplemental Figure 9

Fig.4h

5-LO

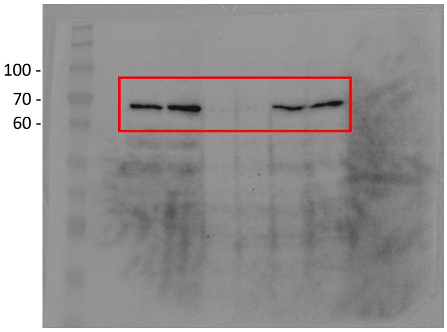

β-actin

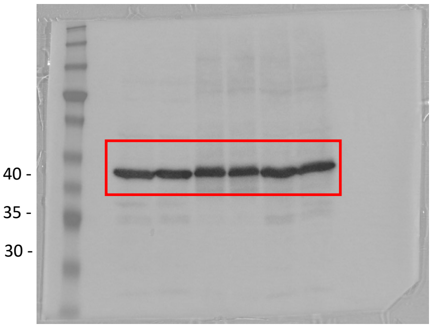

Supplemental Fig.3

5-LO

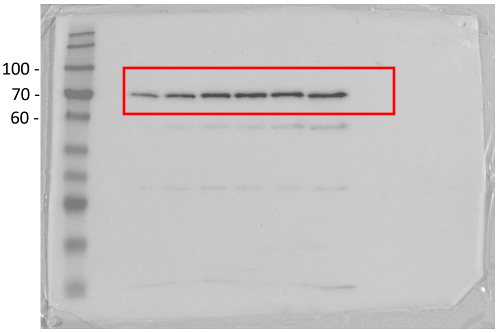

β-actin

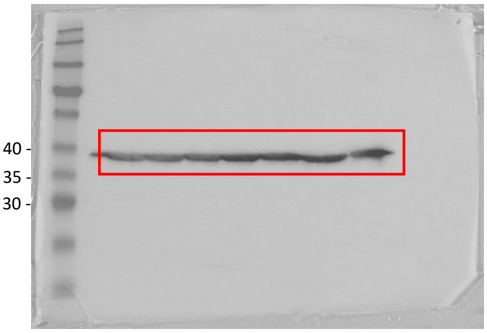

Supplemental Fig.4a

5-LO

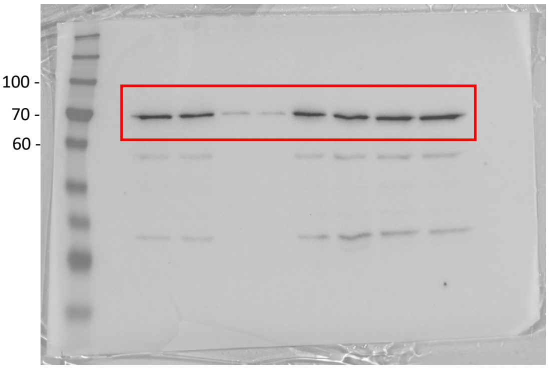

β-actin

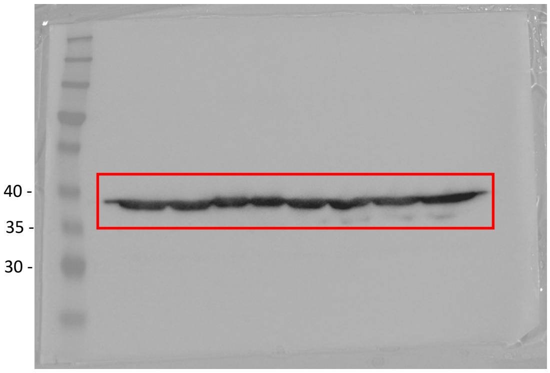

Supplemental Figure 10

Supplemental Fig.4b

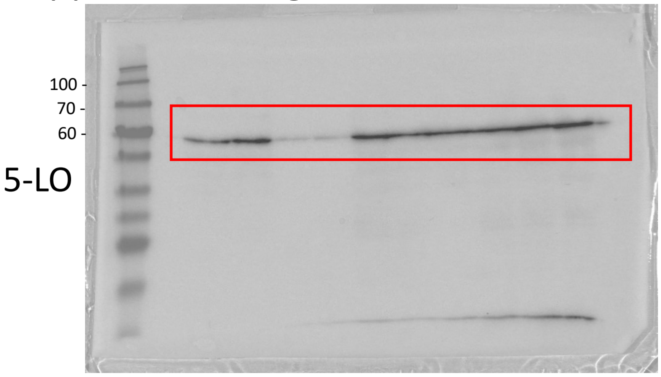

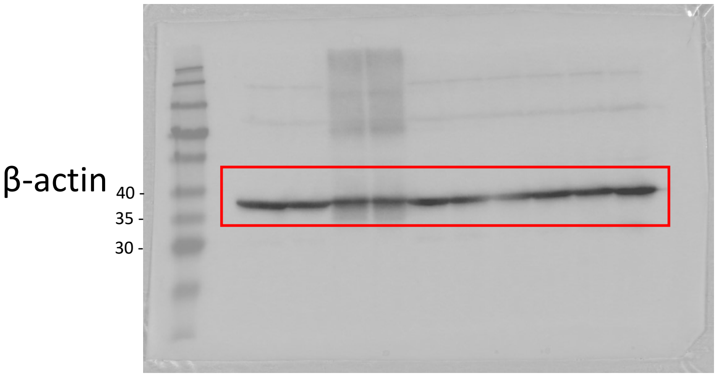

Supplemental Fig.5

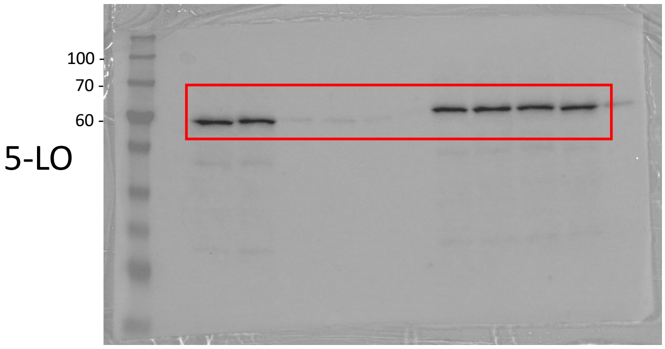

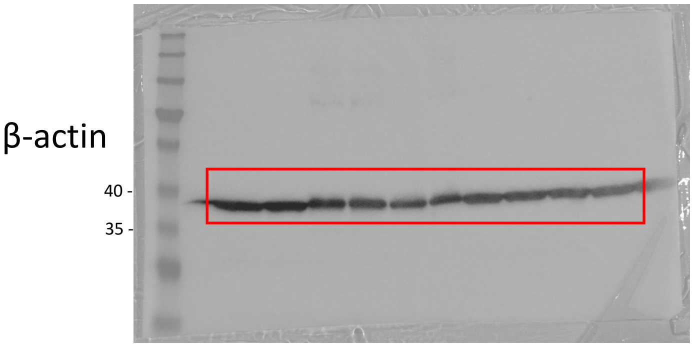

Supplement: Supplementary file 1 — Supplementary Figures. [file 41598_2024_54867_MOESM1_ESM.pdf]
